# Integration of bioeconomy within regional policy frameworks: a case study of the Wielkopolska Voivodeship

**DOI:** 10.5114/bta/205149

**Published:** 2025-06-30

**Authors:** Ewa Woźniak-Gientka

**Affiliations:** Institute of Bioorganic Chemistry Polish Academy of Sciences, Bioeconomy and Sustainable Development Team, Poznan, Poland

**Keywords:** bioeconomy, circular economy, region, Poland, strategy, sustainable development

## Abstract

**Background:**

Recently, many countries and regions have started developing targeted bioeconomy strategies and plans. The concept of the bioeconomy has evolved since the implementation of the first European Union bioeconomy strategy in 2012. Current trends include, among others, the circular economy, biotechnology, and sustainable development.

**Materials and methods:**

This article examines the role of the bioeconomy in the strategic documents of the Wielkopolska Voivodeship, using a case study approach along with analytical and critical methods. Additionally, it explores local government initiatives supporting bioeconomy development, identified through interviews.

**Results:**

In Wielkopolska, there is currently no formal plan to develop a dedicated bioeconomy strategy. However, numerous documents and plans related to the bioeconomy have been developed since 2020, aligning regional policies with the objectives of European Union policy. The local government of Wielkopolska is actively engaged in food waste reduction through education, investment, and support for NGOs and entrepreneurs. There is strong institutional support for bioeconomy-related innovation, including dedicated strategies, funding, competitions, and stakeholder cooperation platforms.

**Conclusions:**

A regional bioeconomy strategy is essential for leveraging local resources, addressing region-specific challenges, and aligning with EU policy frameworks. While Wielkopolska currently lacks a formal strategy, elements of the bioeconomy are included in existing plans. Developing regionally tailored strategies, fostering public awareness, enhancing education, and encouraging cross-sector collaboration is key to building a sustainable, innovation-driven bioeconomy.

## Introduction

The bioeconomy encompasses all sectors and systems that rely on biological resources – including animals, plants, microorganisms, and biomass derived from them, such as organic waste – their functions, and underlying principles. It includes and connects: (1) terrestrial and marine ecosystems and the services they provide; (2) all primary production sectors that use and produce biological resources (such as agriculture, forestry, fisheries, and aquaculture); and (3) all economic and industrial sectors that utilize biological resources and processes to produce food, feed, bio-based products, energy, and services (Bioeconomy Strategy 2018)^i^. Approximately 17.1 million people were employed in the bioeconomy of European Union (EU) countries (EU-27), generating EUR 664 billion in value added. In Poland, 2.42 million people – about 14% of the EU total – worked in the bioeconomy, yet the value added from this sector represented only 6% of the total generated by all EU countries (EUR 38.3 billion) (Knowledge Center for Bioeconomy 2020).

Contemporary global challenges – including climate change, food security, ecosystem degradation, and population growth – have necessitated the development of innovative production and consumption models that respect the ecological limits of the planet (Wozniak et al. 2021). One of the priorities of European policy is to ensure sustainable development across Europe and advance the Sustainable Development Goals (SDGs) introduced by the UN in 2015 (Bioeconomy Strategy 2018; SDGs 2015). However, ongoing conflicts, geopolitical instability, and the COVID-19 pandemic have affected not only the EU economy but also the global economy, emphasizing the vital role of the bioeconomy in future development.

As emphasized by the European Commission (EC), targeted national bioeconomy strategies and/or action plans are crucial for implementing the European Green Deal and generating benefits and opportunities for rural, coastal, regional, and urban areas in each Member State (EC 2021). Poland is among the EU countries that have not yet adopted a national bioeconomy strategy, although it is actively pursuing strategic activities toward its implementation (Haarich and Kirchmayr-Novak 2022). At the national level, a dedicated bioeconomy strategy is currently being developed as part of the BIOEAST Initiative^ii^. The BIOEAST project promotes bioeconomy development across 11 Central and Eastern European countries, including Poland, where implementation remains less advanced. Additionally, Poland has adopted the Circular Economy Roadmap (2019), which encompasses several initiatives aligned with bioeconomy goals. This action plan focuses on sustainable industrial production, sustainable consumption, and the development of new business models.

To date, only one Polish region – Mazovia – has developed a regional bioeconomy strategy (Bioeconomy Development Strategy for the Masovian Voivodeship, 2021). This strategy was created within the framework of the POWER4BIO project^iii^.

In many EU countries, bioeconomy principles are integrated into waste management plans, which promote waste reduction, recycling, and the use of waste for energy production. Elements of the bioeconomy are also present in regional innovation strategies, especially those oriented toward technological advancement, renewable energy, circular economy models, and green growth (Haarich and Kirchmayr-Novak 2022).

This paper aims to examine the role of the bioeconomy in the strategic documents of the Wielkopolska Voivodeship, using it as a case study, and to analyze the initiatives undertaken by local authorities to support its development. Furthermore, the article outlines key components that should be incorporated into a regional strategy to promote the growth of the bioeconomy.

## Theoretical framework

### Conceptual evolution of the Bioeconomy Strategy and initiatives supporting its development

The OECD’s The Bioeconomy to 2030: Designing a Policy Agenda, published in 2009, marked a significant step toward the development of national and regional bioeconomy strategies (OECD 2009). Following this, in 2011, the German Ministry of Education and Research introduced a national strategy titled The National Research Strategy BioEconomy 2030: Our Route Towards a Bio-based Economy, which was later supplemented in 2013 by the National Policy Strategy on Bioeconomy (National Bioeconomy Strategy 2013; Strategy 2011).

In 2012, the EC presented the first European bioeconomy strategy, titled Innovation for Sustainable Growth: A Bioeconomy for Europe (Bioeconomy Strategy 2012). This strategy emphasized the bioeconomy’s role in fostering smart and green growth, aiming to build a more innovative, resource-efficient, and competitive society. It sought to balance food security with the sustainable use of renewable resources for industrial purposes while ensuring environmental protection (Bioeconomy Strategy 2012).

Subsequently, in 2018, the EC published an updated strategy, A Sustainable Bioeconomy for Europe: Strengthening the Connection Between Economy, Society, and the Environment (Bioeconomy Strategy 2018). This update formed part of the EC’s broader efforts to promote employment, economic growth, and investment within the EU. The revised strategy aimed to expand and enhance the sustainable use of renewable resources to tackle both global and local challenges, including climate change and sustainable development. It was projected that the bioeconomy could create one million new green jobs by 2030 (Bioeconomy Strategy 2018).

Since the introduction of the first bioeconomy strategy, the EC has launched numerous initiatives targeting various sectors of the bioeconomy and emphasizing its importance. Notable examples include the European Green Deal (2019), the Circular Economy Action Plan (2020), the Farm to Fork Strategy (2020), the Biodiversity Strategy (2020), the Directive on the Promotion of the Use of Energy from Renewable Sources (2018), and the recently introduced European Wind Power Action Plan (2023). Additionally, the EC has committed to fulfilling the SDGs (SDGs 2015). The implementation of the bioeconomy is crucial for achieving the SDGs; accordingly, recent years have seen growing attention paid to the concept of a sustainable bioeconomy (Liobikiene et al. 2019).

According to the World Commission on Environment and Development (1987), sustainable development is defined as development that meets the needs of the present without compromising the ability of future generations to meet their own needs. This concept encompasses three main dimensions: environmental, economic, and social (Lozano 2008). However, various interpretations exist regarding how sustainable development should be conceptualized (Neto et al. 2018; Ramcilovic-Suominen and Pülzl 2018). Among the most well-known and widely adopted in research are the weak and strong sustainability approaches (Dietz and Neumayer 2007; Liu 2009; Neumayer 2003; Vatn 2009). This understanding of sustainable development can be defined in terms of two orientations: anthropocentric and ecocentric. The anthropocentric approach is essentially associated with the concept of weak sustainability and posits that natural capital and produced capital are perfectly substitutable. This approach implies that economic growth is necessary for environmental protection, and technological innovations can solve all environmental problems. On the other hand, the ecocentric approach is associated with strong sustainability and assumes that the substitutability of natural resources is limited. This means that certain human actions can have irreversible consequences, and environmental protection is necessary for economic growth (Mancebo 2013). Another approach is the biotechnology-oriented bioeconomy, in which economic growth emerges as a central element among the three pillars of the bioeconomy. Facilitating the development of the bioeconomy requires a range of initiatives and funding mechanisms (Liobikiene et al. 2019; Philp 2018; Schütte 2018; Woźniak and Twardowski 2018). From a social perspective, the advancement of biotechnology should contribute to rural development, labor market productivity, wage levels, and employment growth (D’Amato et al. 2017; Schütte 2018).

Furthermore, public perception of biotechnology and the acceptance of bio innovations are critical considerations when introducing biotechnological solutions to ensure sustainable development (Golembiewski et al. 2015; Lynch et al. 2017; Mustalahti 2018). Studies have shown that biotechnologies help mitigate negative environmental impacts, promote the use of renewable resources, and enhance agricultural production efficiency – particularly in food production (Aguilar et al. 2009; Woźniak and Twardowski 2018). Biotechnologies are expected to support the development of economic systems by enabling cleaner and safer production based on renewable resources and recycling (Bell et al. 2018; Lokko et al. 2018; Silveira et al. 2017). In terms of consumption, bioproducts offer a sustainable alternative to those derived from nonrenewable resources (Aguilar et al. 2018; Dupont-Inglis and Borg 2018).

An important issue concerning the development of the bioeconomy and biotechnology is the role of new genomic techniques (NGTs) in EU policy. The EC views NGTs as innovative tools that can enhance the sustainability and resilience of the food system, supporting the goals of the European Green Deal and the Farm to Fork Strategy (EC 2023). This is particularly vital in the context of the current climate crisis and the urgent need to protect the environment and preserve biodiversity. NGTs enable the precise and efficient development of improved plant varieties that can better withstand climatic conditions and pests, require fewer fertilizers and pesticides, or deliver higher yields (EC 2023).

On July 5, 2023, the EC presented a draft proposal concerning plants developed using specific NGTs, outlining detailed and targeted provisions. The proposal applies exclusively to plants produced through targeted mutagenesis and cisgenesis, as well as to related food and feed products. According to Article 3(2) of the proposal, an NGT plant is defined strictly as one obtained by targeted mutagenesis or cisgenesis. Only plants meeting this definition would be eligible for the relaxed provisions of the proposed regulation; however, the regulation also addresses other plants by specifying that they will not benefit from such relaxed provisions (EC 2023). Although approval of the NGT proposal by the European Parliament and the Council may take several years, it represents a significant milestone in the regulatory development of NGTs (EC 2023).

## Materials and methods

The analysis is primarily based on a combination of quantitative and qualitative research methods, particularly those applied in socio-economic geography, economics, and sociology. Additionally, selected sections of the study include statistical evidence provided by the author.

At the end of 2023, meetings and interviews were conducted with seven representatives from several departments of the Marshal Office of the Wielkopolska Region in Poznañ, specifically:

– Department of Economy;
– Department of Regional Policy;
– Department of Agriculture and Rural Development;
– Department of Environmental Management and Climate.

These interviews aimed to gather information about the actions undertaken to support the development of the bioeconomy in the region.

All meetings took place at the Marshal’s Office. The questions and their scope were prepared in advance and shared with the Office representative to ensure a clear understanding of the meeting’s purpose. The participants were selected by a representative of the Office as specialists with extensive knowledge of socio-economic processes in the Wielkopolska region, particularly in the areas of bioeconomy, circular economy, and biotechnology. Moreover, all participants were actively involved in the preparation of documents and development strategies for the voivodeship.

The following methods of data collection and analysis were used:

– Case study: This method involves a detailed, comprehensive description of a single case, allowing for conclusions regarding the causes and effects of its functioning (Stake 1995). In this article, activities supporting the bioeconomy in one Polish region, Wielkopolska, are examined (see Section 4).
– Analytical and critical methods: Strategic documents related to bioeconomy development in the Wielkopolska region were critically reviewed and interpreted (see Section 4.1). The primary criterion was the inclusion of the term bioeconomy in the document’s objectives, as discussed in Section 4.1. In cases where the term was not explicitly mentioned, the author analyzed the content to assess whether it was thematically relevant to the bioeconomy.
– Interview: Meetings were held with representatives of local authorities to determine whether regional efforts are underway to build a bioeconomy strategy, support local bioeconomy producers, and promote the bioeconomy and circular economy within the region. The interview results are presented in Section 4.2.

To strengthen the contextualization of the interviews, a comprehensive analysis of related documentation – including websites, policy reports, and other strategic documents – was also conducted.

## Results

### Place of the bioeconomy in the strategic documents of the Wielkopolska Voivodeship

To date, no dedicated bioeconomy development strategy exists in the Wielkopolska Voivodeship. According to information from the Marshal’s Office, there are currently no plans to develop such a strategy for the region. However, various elements of the bioeconomy are indirectly included in other updated regional strategies, which incorporate provisions from relevant European and national documents in alignment with the current policy perspective. These reference documents include the European Green Deal (2019), the Farm to Fork Strategy (2020), the European Commission’s Hydrogen Strategy for a Climate-Neutral Europe (2020), the Recovery Plan for Europe^iv^, and the Roadmap towards the Transition to the Circular Economy (2019).

The most significant strategic documents in the Wielkopolska Voivodeship that address the bioeconomy through their goals and actions include: – Regional Innovation Strategy for Wielkopolska 2030 (RIS 2030) (2020);

– Development Strategy for the Wielkopolska Voivodeship until 2030 (2020);
– Development Strategy for Eastern Wielkopolska 2040 (2022);
– Climate Neutrality Strategy for Eastern Wielkopolska 2040 (2021);
– Environmental Protection Program for the Wielkopolska Voivodeship until 2030 (2020);
– Wielkopolska Regional Action Plan for Sustainable Energy and Climate in the field of renewable energy sources and energy efficiency with a perspective until 2050 (2021);
– The Strategy for the development of Hydrogen Wielkopolska until 2030 with a perspective until 2040 (2023);
– Air Protection Program for the Wielkopolska Zone (2020);
– Waste Management Plan for the Wielkopolska Voivodeship for 2019–2025 with an investment plan (2020).

The main objective of the RIS 2030 Strategy (2020) is to enhance the innovation and competitiveness of the Wielkopolska Voivodeship through the development of smart specializations. The document identifies six such areas. One of the key specializations relevant to the bioeconomy is “Bioresources and food for conscious consumers,” which encompasses:

– The production of bioproducts and healthy food to ensure food security and cultivate crops resistant to climate change;
– Modern food technologies, biotechnology, and e-agriculture;
– Innovative methods for selling and distributing highquality food, along with the production of food packaging;
– Organic food production and the management of postproduction waste through bioresource generation for other industries, upcycling, and a fuel-energy economy based on agrobiomass.

Another important smart specialization related to the regional bioeconomy is “Industry of Tomorrow”, which includes areas focused on the application of advanced production and special processes, as well as sustainable and efficient energy systems (RIS 2020).

Within the Strategy for the Development of the Wielkopolska Voivodeship until 2030 (2020), several key goals are outlined, including the “development of infrastructure with respect for the natural environment of Wielkopolska”. This goal encompasses operational objectives such as improving the condition and protection of the natural environment, increasing energy efficiency, and enhancing energy safety. From a bioeconomy perspective, significant directions for regional development include increasing and protecting water resources, improving water and air quality, enhancing waste management, preserving biodiversity, improving natural conditions for agriculture, increasing the use of renewable energy sources, and fostering pro-ecological behaviors and attitudes in society.

The strategy also emphasizes the development of emerging sectors driven by global trends – such as the bioeconomy, circular economy, and green economy – as new opportunities for regional growth. Additionally, it highlights the increasing ecological awareness among Europeans and reaffirms that agriculture is, and will remain, one of the fundamental sectors of the region’s economy (Strategy for the Development of the Wielkopolska Voivodeship 2020).

In 2022, the Strategy for the Development of Eastern Wielkopolska 2040 was introduced, reflecting the specific characteristics of the region. The strategy also addressed the impacts of the COVID-19 pandemic and Russia’s aggression against Ukraine, both of which influence the region’s socio-economic development. A key objective in Eastern Wielkopolska is the transition from a coal-based economy to a modern economy built on alternative energy sources, including renewables and hydrogen while promoting sustainable development and respecting social considerations (Strategy for the Development of Wielkopolska 2022).

The strategy also highlights the region’s substantial agricultural and agri-food processing potential, which forms the foundation for bioeconomy development. Among the major challenges identified are the need to foster conditions for a zero-emission and circular economy – encompassing the bioeconomy – increase innovation in agriculture, improve resilience to climate change, and enhance the quality of the natural environment (Strategy for the Development of Eastern Wielkopolska 2022).

The Strategy for Climate Neutrality for Eastern Wielkopolska 2040 (2021) aims to “establish a new pro-climate approach to subregion development and indicate long-term action directions, resulting in the reduction of greenhouse gas emissions and improvement of air quality, development and increased use of renewable energy sources, and reducing the demand for nonrenewable primary energy and increasing energy efficiency” (Climate Neutrality Strategy 2021, p. 8).

The Strategy for the Development of Hydrogen Wielkopolska until 2030 with a Perspective until 2040 (2023) highlights the region’s potential to participate in the hydrogen economy. This potential is supported by favorable conditions for locating renewable energy sources, substantial biogas resources, a well-developed automotive industry, a strategic geographic location at the intersection of key transportation and planned hydrogen transmission routes, as well as the proactive involvement of the local government in the hydrogen sector (e.g., the establishment of the Wielkopolska Hydrogen Platform and the “Hydrogen School” project).

The Environmental Protection Program for the Wielkopolska Voivodeship until 2030 (2020) outlines several goals relevant to the bioeconomy, including climate and air quality protection, water and sewage management, reclamation and revitalization of degraded areas, waste management and prevention, increasing forest cover, biodiversity conservation, and fostering ecological awareness among the population.

The Wielkopolska Regional Action Plan for Sustainable Energy and Climate in the Field of Renewable Sources and Energy Efficiency with a Perspective until 2050 (2021), an update of the 2011 plan, underscores that reducing greenhouse gas emissions in the voivodeship will require substantial financial investment and long-term efforts. The COVID-19 pandemic and the war in Ukraine may impact the condition of enterprises, which may not have sufficient funds to undertake actions to improve energy efficiency. The plan also stresses that developing the hydrogen market for transport could play a major role in reducing emissions from the transport sector in Greater Poland. Additionally, the long-awaited implementation of an agricultural biogas plant construction program could offer a significant source of fuel for public transport in the region (Action Plan 2021).

The most recent documents relevant to the development of the regional bioeconomy include the Air Protection Program for the Wielkopolska Zone (2020) and the Waste Management Plan for the Wielkopolska Voivodeship for the Years 2019–2025, including the accompanying investment plan (2020). The scope of the Air Protection Program aligns with the objectives of bioeconomy development, as improving air quality is essential for enhancing both the quality of life and the health of Wielkopolska’s residents (Air Protection Program 2020). The Waste Management Plan includes a review of forecasts for changes in the quantity of collected and received municipal waste for the years 2017–2030, the processing capacities of municipal installations, as well as plans for the construction and expansion of facilities for processing selectively collected green waste and other biowaste. The updated waste management plan incorporates the regulations of the Circular Economy Package from 2018 (Directive 2018; Waste Management Plan 2020).

### Local government activities toward the development of the bioeconomy: interview results

One of the main activities of the Department of Agriculture and Rural Development of the Marshal’s Office of the Wielkopolska Voivodeship in the area of bioeconomy is the coordination of efforts to reduce food waste at all stages – from production and storage to consumption. These efforts are aligned with the objectives outlined in the Development Strategy for the Wielkopolska Voivodeship. As a result, the Program for Reducing Food Waste and Losses in Wielkopolska for the Years 2021–2025 was adopted by the Board of the Wielkopolska Voivodeship in 2021.

Since the first half of 2021, grant competitions have been organized for nongovernmental organizations and local government units to reduce food waste. These competitions provide financial support for activities such as transporting and storing surplus food, involving aid organizations and food banks. Additionally, they support the creation of food-sharing points, the purchase of small mobile workshop kitchens, and the establishment of food waste reduction and food aid centers in five subregions. By 2023, approximately 3 million PLN had been allocated in the budget of the Wielkopolska Voivodeship Government to combat food waste (Kitchen Without Waste 2023).

Within the Department, activities are carried out on two levels:

1. Educational: These efforts include training sessions, demonstrations (e.g., for the University of the Third Age), cooperation with the Food Bank in Poznañ, and enabling students from gastronomy schools to participate in the Taste of the Region fair. During the most recent fair, the Marshal’s Office covered transportation costs for approximately 600 students. Each year, a wellknown chef is invited to demonstrate how to creatively and effectively use food leftovers.
2. Investment: Food waste reduction centers (4 centers), food transportation and storage facilities (7 nongovernmental organizations), and small mobile kitchens (28) for demonstrations related to waste reduction, along with setting up food-sharing points (28).

The Department also supports local entrepreneurs by organizing exhibition booths at fairs, allowing stakeholders to present their products under the Marshal’s Office banner. Competitions are held for rural women’s circles to further engage community members.

In 2023, a collaborative initiative involving the Wielkopolska Voivodeship Government, the University of Life Sciences, and the Association of the Wielkopolska Food Bank in Poznañ resulted in the publication of a book titled *Kitchen Without Waste: Reducing Food Waste* (2023). The book features a variety of creative recipes developed by students from Wielkopolska who participated in culinary demonstrations and workshops.

An important unit supporting regional innovation is the *Wielkopolska Innovation Observatory*, which operates within the Department of Economy of the Wielkopolska Voivodeship Office in Poznañ. The department’s main tasks include:

– Research and analysis: Identifying the needs of enterprises in Wielkopolska, monitoring changes within smart specialization areas, and addressing regional challenges.
– Monitoring: Tracking the implementation of the Innovation Strategy, the Hydrogen Development Strategy for Wielkopolska, and observing global economic development trends.
– Consultations and recommendations: Conducted in collaboration with the Wielkopolska Smart Specialization Forum and its working groups, aimed at updating innovation policy through the Entrepreneurial Discovery Process.
– Coordination: Overseeing the implementation and promotion of RIS 2030 and organizing initiatives that support the development of smart specialization areas.

The aim and role of the local government are to establish a platform for cooperation among various stakeholders, participate in the *Entrepreneurial Discovery Process*, and develop models of collaboration that support the transition from scientific research to product commercialization. The Wielkopolska Voivodeship Office offers support to entrepreneurs, scientists, and business environment institutions.

In developing a future regional bioeconomy strategy, it is essential to form a team of experts – comprising scientists, entrepreneurs, and representatives of business environment institutions – who will collectively identify actions and directions for advancing the bioeconomy in the region. The Wielkopolska Voivodeship Office actively collaborates with various stakeholders, including through established Smart Specialization Working Group Forums, such as the “Biomass and Food Working Group for Conscious Consumers”.

The Department of Economy also plays a role in promoting regional brands in global and European markets by enabling local entrepreneurs to showcase their products at food fairs. Additionally, it prepares attractive offers tailored to startups. The Wielkopolska Voivodeship Office has partnered with several Business Environment Institution incubation programs, including those run by the Turkish Chamber of Commerce, Prometeia, the Association for Supporting Entrepreneurship of the Gostyñ County, the Leszno Business Center, You Nick, the Wielkopolska Eco-Entrepreneurship Incubator, *All For Innovators – Business Development Program from Wielkopolska*, and CD Centrum Business Environment Institution.

An important opportunity for entrepreneurs in the Wielkopolska region is participation in the competition organized by the Wielkopolska Voivodeship Office, “i-Wielkopolska – Innovations for Wielkopolska”^v^. In this competition, companies introducing innovative solutions to the market are recognized in two categories: “Smart Specializations for Wielkopolska” and “H2 Greater Poland.” All business entities operating in the Wielkopolska Voivodeship are eligible to participate. Winners receive cash prizes and promotional support during economic events organized by the Wielkopolska Voivodeship Office. The local government also supports the scientific sector by organizing the competition for the Marshal of the Wielkopolska Voivodeship Award “Wielkopolska for the Planet 2030”^vi^. This competition is open to PhD holders, doctoral students, and regional enterprises. The aim is to promote the region’s contribution to achieving SDGs and to promote knowledge transfer to the economy, enabling real changes that incorporate pro-climate solutions in all areas of socio-economic life.

Representatives of the Wielkopolska Voivodeship Office also promote the region on the European stage and organize conferences and meetings for interested parties. Furthermore, the Office provides financial support through European Funds for Wielkopolska for the years 2021–2017^vii^. For the development of the bioeconomy and biotechnology in the region, activities such as: supporting the transformation toward a circular economy and resource-efficient economy, supporting the R&D potential of research entities in the region and development of renewable energy sources are of particular importance.

The Department of Environmental and Climate Management is primarily responsible for issuing permits for the introduction of substances or energy into the environment and for granting concessions related to mining activities. It also issues decisions concerning waste management. The department prepares strategic documents addressing environmental protection, air quality, environmental noise, waste management, and asbestos removal.

In addition, the department carries out activities related to postimplementation analyses, ecological reviews, the designation of areas of limited use, and actions focused on climate protection. These include both the mitigation of climate change effects and adaptation strategies.

## Discussion

### What key elements should be included in the development of a bioeconomy strategy?

The presence of regional strategies related to the bioeconomy largely depends on two key factors. First, in large and decentralized countries, regional bioeconomy strategies are more likely to exist. Second, if a national bioeconomy strategy is in place, it may guide regional and local actions, thereby reducing the need for separate regional strategies. In such cases, bioeconomy-related objectives may be embedded within broader strategic frameworks at subnational levels. Nonetheless, even when a national strategy exists, regional frameworks may still emerge to define specific actions and address regional specificities^viii^.

In 2022, there were 359 bioeconomy-related strategies in the EU. Of the 334 published documents, 324 were regional in scope, and 10 represented multiregional strategic frameworks (e.g., cross-border, inter-regional, or macroregional) (Haarich and Kirchmayr-Novak, 2022). Approximately 77% of all strategies identified the sustainable management of natural resources as a primary objective. Reducing reliance on nonrenewable resources was also a frequently cited goal, present in 68% of strategies. Many strategies – especially those integrating bioeconomy themes into smart specialization or economic frameworks – emphasized the enhancement of regional competitiveness and job creation (63%). Additionally, the majority of strategies (57%) aligned with Europe’s overarching objective of mitigating and adapting to climate change, often in connection with the use of bio-based resources. Ensuring food security appeared less frequently, identified as a key goal in only 27% of strategies (Haarich and Kirchmayr-Novak 2022).

In Poland, 32 regional strategies related to the bioeconomy have been identified, including one explicitly dedicated to its development: the draft Bioeconomy Development Strategy for the Masovian Voivodeship. Among the 31 other strategic frameworks, 16 were sectoral strategies – 14 in the waste sector, one in agriculture/agri-food, and one in energy. The bioeconomy was also included in 15 broader strategic frameworks: eight research and innovation strategies and seven regional or territorial development strategies (Haarich and Kirchmayr-Novak 2022; Masovian Strategy 2021). Several strategic documents relevant to the bioeconomy were also identified in Wielkopolska, as detailed in Section 4.1.

An essential component of bioeconomy development is the publication and dissemination of research findings, as well as the sharing of best practices in the field. The Wielkopolska Voivodeship possesses significant research potential through its scientific and R&D institutions. Notable institutions benefiting from EU research funding include the Institute of Bioorganic Chemistry of the Polish Academy of Sciences, Poznañ University of Technology, and the £ukasiewicz Research Network.

Best practices suggest that the most effective approach to developing the bioeconomy is through the design and implementation of national strategies and/or action plans, supported by active stakeholder engagement and accompanied by strong implementation and monitoring frameworks (EC 2021). Among countries demonstrating exemplary high-level political support is Italy, where the government launched a national bioeconomy strategy in 2017, later updated in 2019 to better integrate the core pillars of the national bioeconomy. The updated strategy focuses on enhancing coordination among ministries and Italian regions in adapting policies, regulations, funding programs for research and innovation, and infrastructure investments. Its objective is to increase turnover and employment in the Italian bioeconomy by 15% by 2030 (BIT II 2019; Fava et al. 2021). Countries with exemplary practices in terms of fairness, openness, and inclusion of various stakeholders include Austria and Germany. In Austria, an important element of developing strategies was involving stakeholders and establishing an interdisciplinary advisory group, the bioeconomy platform (Austria Strategy 2019). In the German bioeconomy strategy (National Bioeconomy Strategy 2020), the need to use external specialized knowledge was emphasized. During the consultation process, feedback and recommendations were provided by 16 state governments, the German Bioeconomy Council, four scientific institutions, 17 industry associations, and 15 NGOs working in environmental protection, development, and consumer advocacy (EC 2021).

At the EU level, numerous regional bioeconomy strategies have also been developed. In Germany alone, six regions have published strategies fully dedicated to the bioeconomy (EC 2021).

Regional bioeconomy strategies are crucial for improving living conditions in urban and rural areas and for sustainably managing natural resources. Achieving a sustainable future requires both a global perspective – through the creation of international governance frameworks – and local action, including public education, awareness campaigns, stakeholder engagement, and collaborative projects. Long-term thinking must also be prioritized. Increasing awareness and knowledge about the bioeconomy should begin at the local level and actively involve younger generations. Therefore, mobilizing local and regional communities and fostering their understanding of the bioeconomy is crucial. Environmental awareness surveys conducted by the Wielkopolska Spatial Planning Office in Poznañ, involving 1,200 residents, highlighted the importance of the natural environment to the people of Wielkopolska – 84% of respondents considered it important. However, 64% of respondents evaluated the state of the environment negatively, while only 29% rated it positively. Two-thirds of respondents believed that the state of the natural environment depends on the actions of each individual. Furthermore, residents believed that the environment’s state hinges on society’s recognition of environmental issues as important (41%), the government’s emphasis on environmental issues (38%), the formulation and enforcement of appropriate legal regulations (36%), and the activities of local authorities in environmental protection (32%). Respondents indicated that both individuals and government authorities play roles in shaping ecological attitudes and behaviors, with 60% and 58% of respondents attributing responsibility to them, respectively^x^. Residents of the Wielkopolska demonstrated awareness of ongoing climate change (96% of respondents). About 88% of respondents believed that residents should take action to minimize the adverse effects of climate change, while 50% pointed to central government authorities^xi^.

A strategy or action plan for developing the regional bioeconomy should include a concept that improves the organization of education and the dissemination of knowledge – particularly among the general population, youth, and children. This may involve organizing workshops, webinars, thematic meetings, and informational campaigns conducted throughout the region. At the national level, it is equally important to align educational programs with current development trends. Such alignment will not only enhance students’ knowledge in areas such as the bioeconomy, circular economy, recycling, eco-design principles, and sustainable development but also prepare them for future employment in emerging industries.

Several universities in Poznañ offer courses related to the bioeconomy. These include Adam Mickiewicz University^xii^ (e.g., Faculty of Chemistry, Biology, and Physics), the University of Life Sciences^xiii^ (e.g., Faculty of Agriculture and Bioengineering, Faculty of Forestry, Faculty of Food and Human Nutrition, Faculty of Wood Technology), Karol Marcinkowski Medical University^xiv^ (Medical Biotechnology), and Poznañ University of Technology^xv^ (e.g., Faculty of Civil and Environmental Engineering, Faculty of Chemical Technology, Faculty of Bioinformatics).

The transition to a sustainable, closed-loop bioeconomy in the Member States will require the involvement of numerous entities over extended periods, both within and beyond government structures. To enable such engagement, it is essential to create space for collaboration in identifying, designing, and implementing a shared vision for the bioeconomy. An illustrative example is the *BIOEAST HUB CZ* in the Czech Republic, which brings together more than 100 stakeholders – including research institutions, technological platforms, companies, and NGOs (EC 2021)^xvi^.

Transitioning to a closed-loop bioeconomy often extends beyond the jurisdiction of any single government department. A key challenge lies in building collective leadership capacity to introduce innovations across the entire government. This requires integrating bioeconomy principles into multiple policy areas, such as entrepreneurship, environmental and climate policy, the circular economy, agriculture, fisheries, forestry, scientific research, innovation, and education (EC 2021).

Transitioning to a sustainable, closed-loop bioeconomy will also require behavioral changes at the individual level, including among raw material producers, consumers, and society as a whole (Fritsche et al. 2021; Gaffey et al. 2021). Communication activities are therefore essential, involving active engagement and actions with all interested parties (Kelleher et al. 2021).

At the national level, a bioeconomy strategy should include provisions to eliminate legal loopholes – such as those affecting the operation of biogas plants. The strategy should also address the need for easily accessible financial support for investors and establish a streamlined process for obtaining the necessary administrative approvals for biogas plant construction^xvii^.

In 2022, the Wielkopolska Voivodeship had 35 biogas installations^xviii^, making it one of the leading regions in terms of biogas production potential. With its extensive agricultural land and a well-developed agri-food sector, the region has strong potential for the further development of agricultural biogas plants. However, the Polish biogas and biomethane market faces administrative barriers that have effectively limited the sector’s development to date. Proposed regulations that liberalize the functioning of biogas plants may represent a new opportunity for the industry.

Moreover, a future bioeconomy strategy should define clear conditions for better organizing and distributing biomass resources, thereby contributing to more efficient and resilient supply chains (Environmental Protection Program 2020; Wielkopolska Hydrogen Development Strategy 2023; Woźniak 2015).

A comprehensive bioeconomy policy must also align with broader environmental and waste management objectives. One critical area requiring immediate attention is the implementation of European Union directives concerning waste. Directive (EU) 2018/851, which amends the Waste Framework Directive, introduced the concept of Extended Producer Responsibility (EPR) across the EU. However, EPR has not yet been transposed into Polish law, despite the legislative deadline expiring on January 5, 2023. Under EPR, producers placing products on the market are financially and organizationally responsible for the product throughout its lifecycle, up to the point it becomes waste. The prolonged legislative work on this issue primarily has a negative impact on residents, as they bear all the costs of operating municipal waste management systems, and also on recycling companies^xix,xx,xxi^. Beyond appropriate legal frameworks, addressing mental and institutional barriers is also necessary. Enhancing understanding of waste management principles among municipal authorities, entrepreneurs, and the wider public is key to achieving effective outcomes.

Another important component of the regional strategy is the establishment of principles for monitoring bioeconomy development. It is essential to define indicators that can be used to assess the state of the bioeconomy in the region and allow for comparison with other regions. This requires filling existing gaps in statistical data sources. Special attention should be paid to the classification of bioeconomy-related indicators, including the potential for distinguishing circular economy and bioeconomy indicators at the NUTS 2 and NUTS 3 levels. The overarching goal is to implement and integrate various databases and leverage their contents to demonstrate to the public the progress being made toward achieving strategic bioeconomy development goals (Gardossi et al. 2023).

## Conclusion and recommendations

A regional bioeconomy development strategy is not only desirable but essential for the effective utilization of local resources and potential. Given Poland’s considerable economic and environmental diversity, a uniform national approach is insufficient. Regional strategies make it possible to tailor actions to the unique characteristics of each area – its natural resources, environmental challenges, and economic structure. They also facilitate access to EU funding, support innovative projects, and help establish local value chains grounded in the bioeconomy. A well-designed strategy can serve as a catalyst for building a sustainable, low-emission, and competitive regional economy.

Currently, there are no formal plans to develop a dedicated bioeconomy strategy in the Wielkopolska Voivodeship. Nevertheless, many regional strategies and plans contain objectives related to key areas of the bioeconomy, including renewable energy, waste management, hydrogen technologies, and more. Notably, most of these strategies have been developed since 2020, reflecting the regional authorities’ commitment to aligning local policies with the priorities outlined in major national and EU strategic documents.

The recommendations presented below outline key elements important for the development of the bioeconomy in the region. They are not intended to be exhaustive but aim to propose and highlight potential directions for advancing the present and future bioeconomy in the region. The necessary elements relevant to the development of a regional bioeconomy strategy are:

1. Determining the potential of the bioeconomy in individual regions and then defining the goals and directions of activities for the development of the bioeconomy in the region (in accordance with the message that each region has its own potential); striving to create separate regional strategies or bioeconomy action plans – the choice should depend on the capabilities of a given region and local government authorities;
2. Using good practices introduced by other countries and regions;
3. Public education and acceptance of new innovative solutions by the region’s inhabitants;
4. Social activities increasing residents’ awareness of the development of the bioeconomy;
5. Development of human capital, further increase in education in biotechnology, engineering, and agricultural fields (education of future specialists);
6. At the national level, changing the school curriculum – greater attention to issues related to the bioeconomy: sustainable development, circular economy, recycling, biodiversity, etc.;
7. Strengthening cooperation between entrepreneurs from the biotechnology industry, local/regional governments, industry associations, and scientific and research and development units;
8. Establishing provisions facilitating the operation of biogas plants and introducing provisions on EPR at the national level;
9. Monitoring the development of the bioeconomy; determining indicators of bioeconomy development in the region.

The recommendations outlined above directly address the theoretical foundations and challenges discussed in the initial sections. By integrating both strong sustainability principles and the biotechnologyoriented approach to the bioeconomy, the proposed actions aim to strike a balance between environmental protection and economic and technological advancement. Initiatives such as enhancing public education, promoting social acceptance of innovation, and developing human capital align with the goal of fostering ecocentric, socially inclusive bioeconomic growth. Moreover, measures related to policy development, stakeholder cooperation, and monitoring mechanisms underscore the importance of building a resilient, regionally adapted framework that supports sustainable innovation – particularly in response to evolving EU policies on biotechnology and new genomic techniques.

## Resources

i
https://knowledge4policy.ec.europa.eu/bioeconomy/bioeconomy--strategy_en

ii
https://bioeast.eu/

iii
https://power4bio.eu/

iv
https://www.consilium.europa.eu/pl/policies/eu-recovery-plan/

v
http://iw.org.pl/iwielkopolska/

vi
https://www.umww.pl/konkurs-wielkopolska-dla-planety-2030

vii
https://wrpo.wielkopolskie.pl/dowiedz-sie-wiecej-o-programie/fundusze-europejskie-dla-Wielkopolski-2021-2027

viii
https://joint-research-centre.ec.europa.eu/jrc-news-and-updates/bioeconomy-strategies-eu-regions-where-are-we-2022-10-13_en

x https://wbpp.poznan.pl/133/swiadomosc-ekologiczna-mieszkancow-wojewodztwa-wielkopolskiego-wyniki-badania-cz-1.html)

xi
https://wbpp.poznan.pl/147/swiadomosc-ekologiczna-mieszkancow-wojewodztwa-wielkopolskiego-wyniki-badania-cz-2.html

xii
https://amu.edu.pl/

xiii
https://puls.edu.pl/kandydat/kierunki-studiow-2s

xiv
https://www.ump.edu.pl/kierunki-ksztalcenia

xv
https://www.put.poznan.pl/pl/studia-i-oraz-ii-stopnia/oferta-edukacyjna-kierunki-studiow

xvi
https://www.bio-hub.cz/en/

xvii
https://www.parkiet.com/energetyka/art37747391-polski-rynek--biogazu-ma-ogromny-potencjal-i-bariery

**xviii:** https://www.ure.gov.pl/pl/oze/potencjal-krajowy-oze/8108,Instalacje-odnawialnych-zrodel-energii-wg-stanu-na-dzien-31-grudnia-2018-r.html

xix
https://ecoekonomia.pl/2023/09/21/recyklerzy-zadaja-wyjasnien--od-premiera-w-sprawie-rop/

xx
https://ecoekonomia.pl/2022/12/07/rop-2/

xxi
https://www2.deloitte.com/pl/pl/pages/tax/articles/Czym-jest-Rozszerzona-Odpowiedzialnosc-Producenta-ROP-i-jakie-sa-planowane-zmiany-ws-ROP.html

## References

[cit0001] Action (Plan 2021). Marshal Office of the Wielkopolska Region. [In Polish]. https://arrtransformacja.org.pl/wp-content/uploads/2021/03/3113_zalacznik.pdf [accessed 20 May 2024]

[cit0002] Aguilar A, Bochereau L, Matthiessen L. 2009. Biotechnology as the engine for the knowledge-based bio-economy. Biotechnol Genet Eng Rev. 26(1): 371–388. 10.5661/bger-26-371 21415889

[cit0003] Aguilar A, Wohlgemuth R, Twardowski T. 2018. Perspectives on bioeconomy. New Biotechnol. 40: 181–184. 10.1016/j.nbt.2017.06.012 28711519

[cit0004] Air Protection Program. 2020. Marshal Office of the Wielkopolska Region. Contractor: Biuro Studiów i Pomiarów Proekologicznych „EKOMETRIA” Sp. z o.o. [In Polish]. https://bip.umww.pl/artykuly/2826214/pliki/20200715161251_391.pdf [accessed 25 May 2024]

[cit0005] A new circular economy action plan for a cleaner and more competitive Europe. 2015. EC, 98, Brussels, Belgium.

[cit0006] Austria Strategy. 2019. Federal Ministries of Republic of Austria – Sustainability and Tourism; Education, Science and Research; Transport, Innovation and Technology. Bioeconomy – A Strategy for Austria. https://www.bmk.gv.at/en/topics/climate-environment/climate-protection/bioeconomy-strategy.html

[cit0007] Bell J, Paula L, Dodd T, Németh S, Nanou C, Mega V, Campos P. 2018. EU ambition to build the world’s leading bioeconomy – Uncertain times demand innovative and sustainable solutions. New Biotechnol. 40: 25–30. 10.1016/j.nbt.2017.06.010 28676417

[cit0008] BIT II. 2019. Bioeconomy in Italy: A new bioeconomy strategy for a sustainable Italy. https://knowledge4policy.ec.europa.eu/publication/bit-ii-bioeconomy-italy-new-bioeconomy-strategy-sustainable-italy_en

[cit0009] Bioeconomy development strategy for the Masovian Voivodeship. 2021. [In Polish]. Masovian Energy Agency project. https://www.mae.com.pl/images/PDF/POWER4BIO/Strategia_rozwoju_biogospodarki_dla_Wojew%C3%B3dztwa_Mazowieckiego.pdf [accessed 30 May 2024]

[cit0010] Bioeconomy Strategy. 2018. A sustainable Bioeconomy for Europe: Strengthening the connection between economy, society, and the environment. COM, 673 final. Brussels, Belgium.

[cit0011] Bioeconomy Strategy. 2012. Innovating for sustainable growth: a bioeconomy for Europe. European Commission, Brussels, Belgium.

[cit0012] Biodiversity Strategy for 2030. 2020. Bringing nature back into our lives. EC, 380, Brussels, Belgium.

[cit0013] Climate Neutrality Strategy for Eastern Wielkopolska 2040. 2021. Marshal Office of the Wielkopolska Region. [In Polish]. Contractor: Wielkopolskie Biuro Planowania Przestrzennego w Poznaniu. https://arrtransformacja.org.pl/wp-content/uploads/2021/03/1_SNK_11_03_2021.pdf [accessed 25 May 2024]

[cit0014] D’Amato D, Droste N, Allen B, Kettunen M, Lähtinen K, Korhonen J, Leskinen P, Matthies BD, Toppinen A. 2017. Green, circular, bio economy: A comparative analysis of sustainability avenues. J Clean Prod. 168: 716–734. 10.1016/j.jclepro.2017.09.053

[cit0015] Development Strategy for Eastern Wielkopolska 2040. 2022. [In Polish]. Contractor: ARR Transformacja Sp. z o.o. under the supervision of M. Sytka and Wielkopolskie Biuro Planowania Przestrzennego in Poznan under the supervision of J. Maækowiak. https://wbpp.poznan.pl/185/strategia-rozwoju-wielkopolski-wschodniej-2040.html [accessed 15 June 2024]

[cit0016] Development Strategy for the Wielkopolska Voivodeship until 2030. 2020. Marshal Office of the Wielkopolska Region. [In Polish]. https://bip.umww.pl/292---k_91---k_207---strategia-rozwoju-wojewodztwa-wielkopolskiego-do-2030 [accessed 30 May 2025]

[cit0017] Dietz S, Neumayer E. 2007. Weak and strong sustainability in the SEEA: Concepts and measurement. Ecol Econ. 61: 617–626. 10.1016/j.ecolecon.2006.09.007

[cit0018] Directive (EU) 2018/851 of the European Parliament and of the Council of 30 May 2018 amending Directive 2008/98/EC on waste.

[cit0019] Directive (EU) 2018/2001 of the European Parliament and of the Council of 11 December 2018 on the promotion of the use of energy from renewable sources, L 328/82.

[cit0020] Dupont-Inglis J, Borg A. 2018. Destination bioeconomy – The path towards a smarter, more sustainable future. New Biotechnol. 40: 140–143. 10.1016/j.nbt.2017.05.010 28587885

[cit0021] EC. 2023. Proposal for a regulation of the European Parliament and of the council on plants obtained by certain new genomic techniques and their food and feed, and amending Regulation (EU) 2017/625. COM(2023) 411 final. Brussels.

[cit0022] EC. 2021. Deploying the Bioeconomy in the EU: A framework approach for bioeconomy strategy development. 10 policy recommendations for building national bioeconomies toward a fair and just climate neutral Europe.

[cit0023] European Green Deal. 2019. Communication from the commission to the European parliament, the council, the european economic and social committee and the committee of the regions. COM(2019), 640 final. Brussels, Belgium.

[cit0024] European Wind Power Action Plan. 2023. COM(2023) 669 final, Brussels, Belgium.

[cit0025] Farm to fork strategy. 2020. Strategy for a fair, healthy and environmentally-friendly food system. EC, 381, Brussels, Belgium.

[cit0026] Fava F, Gardossi L, Brigidi P, Morone P, Carosi DAR, Lenzi A. 2021. The bioeconomy in Italy and the new national strategy for a more competitive and sustainable country. New Biotechnol. 61: 124–136. 10.1016/j.nbt.2020.11.009 33220517

[cit0027] Fritsche U, Brunori G, Chiaramonti D, Galanakis C, Matthews R, Panoutsou C. 2021. Future transitions for the Bioeconomy towards Sustainable Development and a Climate-Neutral Economy – Foresight Scenarios for the EU bioeconomy in 2050. In: Borzacchiello MT, Stoermer E, Avraamides M (Eds.). Publications Office of the European Union, Luxembourg. ISBN 978-92-76-28413-0, 10.2760/763277

[cit0028] Gaffey J, McMahon H, Marsh E, Vehmas K. 2021. Understanding consumer perspectives of bio-based products – A Comparative Case Study from Ireland and The Netherlands. Sustainability 13: 6062. 10.3390/su13116062

[cit0029] Gardossi L, Philp J, Fava F, Winickoff D, D'Aprile L, Dell'Anno B, Marvik OJ, Lenzi A. 2023. Bioeconomy national strategies in the G20 and OECD countries: Sharing experiences and comparing existing policies. EFB Bioecon J. 3: 100053. https://www.sciencedirect.com/science/article/pii/S2667041023000083

[cit0030] Golembiewski B, Sick N, Bröring S. 2015. The emerging research landscape on bioeconomy: What has been done so far and what is essential from a technology and innovation management perspective? Innovative Food Sci Emerg Technol. 29: 308–317. 10.1016/j.ifset.2015.03.006

[cit0031] Haarich S, Kirchmayr-Novak S. 2022. Bioeconomy strategy development in EU regions. In: Sanchez Lopez J, Borzacchiello MT, Avraamides M (Eds.). Publications Office of the European Union, Luxembourg. ISBN 978-92-76-50040-7, 10.2760/15613

[cit0032] Kelleher L, Henchion M, O’Neill E. 2021. Framing the Circular Bioeconomy in Ireland’s Broadsheet Media, 2004–2019. Environ Commun. 15(5): 678–698. 10.1080/17524032.2021.1889632

[cit0033] Kitchen Without Waste. 2023. [In Polish]. Kuchnia bez strat, czyli ograniczamy marnowanie żywności. Marshal Office of the Wielkopolska Region.

[cit0034] Knowledge Centre for Bioeconomy. 2020. https://knowledge4policy.ec.europa.eu/bioeconomy/topic/economy_en [accessed 18 Jan 2024].

[cit0035] Liobikiene G, Balezentis T, Streimikiene D, Chen X. 2019. Evaluation of bioeconomy in the context of strong sustainability. Sustain Dev. 27(5). 10.1002/sd.1984

[cit0036] Liu L. 2009. Sustainability: Living within one’s own ecological means. Sustainability 1(4): 1412–1430. 10.3390/su1041412

[cit0037] Lokko Y, Heijde M, Schebesta K, Scholtès P, van Montagu M, Giacca M. 2018. Biotechnology and the bioeconomy — Towards inclusive and sustainable industrial development. New Biotechnol. 40: 5–10. 10.1016/j.nbt.2017.06.005 28663120

[cit0038] Lozano R. 2008. Envisioning sustainability three-dimensionally. J Clean Prod. 16: 1838–1846. 10.1016/j.jclepro.2008.02.008

[cit0039] Lynch DHJ, Klaassen P, Broerse JEW. 2017. Unraveling Dutch citizens’ perceptions on the bio-based economy: The case of bioplastics, bio-jetfuels and small-scale bio-refineries. Ind Crops Prod. 106: 130–137. 10.1016/j.indcrop.2016.10.035

[cit0040] Mancebo F. 2013. Développement durable (2ème édition ed.). Paris: Armand Colin.

[cit0041] Mustalahti I. 2018. The responsive bioeconomy: The need for inclusion of citizens and environmental capability in the forest based bioeconomy. J Clean Prod. 172: 3781–3790. 10.1016/j.jclepro.2017.06.132

[cit0042] National Bioeconomy Strategy. 2013. The Federal Government. Germany.

[cit0043] National Bioeconomy Strategy. 2020. German Federal Ministry of Education and Research and Federal Ministry of Food and Agriculture. https://www.bmbf.de/en/bioeconomy---new-concepts-for-the-utilization-of-natural-resources-4543.html

[cit0044] Neto GCO, Pinto LFR, Amorim MPC, Giannetti BF, Almeida CMVB. 2018. A framework of actions for strong sustainability. J Clean Prod. 196: 1629–1643. 10.1016/j.jclepro.2018.06.067

[cit0045] Neumayer E. 2003. Weak versus strong sustainability: Exploring the limits of two opposing paradigms. Cheltenham, UK: Edvard Elgar.

[cit0046] OECD. 2009. The Bioeconomy to 2030: designing a policy agenda. https://www.oecd.org/sti/futures/long-termtechnologicalsocietalchallenges/thebioeconomyto2030designingapolicyagenda.htm [accessed 18 Jan 2024].

[cit0047] Philp J. 2018. The bioeconomy, the challenge of the century for policy makers. New Biotechnol. 40: 11–19. 10.1016/j.nbt.2017.04.004 28487094

[cit0048] Ramcilovic-Suominen S, Pülzl H. 2018. Sustainable development – A ‘selling point’ of the emerging EU bioeconomy policy framework? J Clean Prod. 172: 4170–4180. 10.1016/j.jclepro.2016.12.157

[cit0049] Regional Innovation Strategy for Wielkopolska 2030 RIS. 2020. Marshal Office of the Wielkopolska Region. [In Polish]. Contractor: Innoreg Sp. z o.o. https://www.umww.pl/artykuly/54802/pliki/20210104081530_ris2030.pdf [20 May 2024].

[cit0050] Roadmap towards the Transition to Circular Economy. 2019. The Council of Ministers. Resolution No 136/2019. https://circulareconomy.europa.eu/platform/en/strategies/polands-circular-economy-roadmap [accessed 10 May 2024].

[cit0051] Schütte G. 2018. What kind of innovation policy does the bioeconomy need? New Biotechnol. 40: 82–86. 10.1016/j.nbt.2017.04.003 28458016

[cit0052] SDGs. 2015. Transforming our World: The 2030 Agenda for Sustainable Development. A/RES/70/1. United Nations.

[cit0053] Silveira S, Khatiwada D, Leduc S, Kraxner F, Venkata BK, Tilvikine V, Kalinichenko A. 2017. Opportunities for bioenergy in the Baltic Sea Region. Energy Proc. 128: 157–164. 10.1016/j.egypro.2017.09.036

[cit0054] Stake RE. 1995. The art of case study research. Thousand Oaks, CA: Sage.

[cit0055] Strategy. 2011. National Research Strategy BioEconomy 2030: Our Route towards a Biobased Economy. Bonn, Berlin: Federal Ministry of Education and Research.

[cit0056] The Environmental Protection Program for the Wielkopolska Voivodeship until 2030. 2020. [In Polish]. Contractor: EKOSTANDARD Pracownia Analiz Środowiskowych. Authors: Siudak R, Płaza M, Pawlicki K, Garbacz J, Helińska K. https://bip.umww.pl/292---555---k_91---k_93---programu-ochrony-srodowiska-dla-wojewodztwa-wielkopolskiego [accessed 20 May 2024]

[cit0057] The European Commission’s Hydrogen Strategy for a Climate-Neutral Europe. 2020. COM(2020) 301. Brussels, Belgium. Communication from the Commission to the European Parliament, the Council, the European Economic and Social Committee and the Committee of the Regions.

[cit0058] The Strategy for the development of hydrogen Wielkopolska until 2030, with a perspective until 2040. 2023. Marshal Office of the Wielkopolska Region. [In Polish]. Contractor: NEXUS Consultants sp. z o.o. Majewski W, Foltynowicz M, Pelc TF, Siatkowski M (Eds.). Available from http://iw.org.pl/wp-content/uploads/2023/06/The-Strategy-forthe-development-of-hydrogen-Wielopolska-until-2030-witha-perspective-until-2040-summary.pdf [30 May 2024]

[cit0059] The Waste Management Plan for the Wielkopolska Voivodeship for the years 2019–2025 along with the investment plan. 2020. [In Polish]. https://bip.umww.pl/292---555---k_91---k_92---informacja-o-przyjeciu-planu-gospodarki-odpadami [25 May 2024]

[cit0060] The World Commission on Environment and Development. 1987. Our Common Future. United Nations.

[cit0061] Tyczewska A, Twardowski T, Woźniak-Gientka E. 2023. Agricultural biotechnology for sustainable food security. Special issue: 40^th^ anniversary. Trends Biotechnol. 41(3): 331–341. 10.1016/j.tibtech.2022.12.013 36710131 PMC9881846

[cit0062] Woźniak E. 2015. Renewable energy sources in Wielkopolska. Analysis of deployment of biogas power plant, biomass and co-firing. [In Polish]. Rozwój Regionalny i Polityka Regionalna 32: 137–147.

[cit0063] Woźniak E, Tyczewska A. 2021. Bioeconomy during the COVID-19 and perspectives for the post-pandemic world: Example from EU. EFB Bioecon J. 1: 100013. 10.1016/j.bioeco.2021.100013

[cit0064] Woźniak E, Twardowski T. 2018. The bioeconomy in Poland within the context of the European Union. New Biotechnol. 40: 96–102. 10.1016/j.nbt.2017.06.003 28647548

[cit0065] Woźniak E, Tyczewska A, Twardowski T. 2021. A shift towards biotechnology: Social opinion in the EU. Trends Biotechnol. 39(3): 214–218. 10.1016/j.tibtech.2020.08.001 32896439

[cit0066] Vatn A. 2009. Sustainability, institutions and behaviour. In: Beckmann V, Padmanabhan M (Eds.). Institutions and sustainability. Dordrecht: Springer, Chapter 14, pp. 293–314. 10.1007/978-1-4020-9690-7_14

